# Simulating the Cortical 3D Visuomotor Transformation of Reach Depth

**DOI:** 10.1371/journal.pone.0041241

**Published:** 2012-07-16

**Authors:** Gunnar Blohm

**Affiliations:** Centre for Neuroscience Studies, Queen's University, Kingston, Ontario, Canada; University of Muenster, Germany

## Abstract

We effortlessly perform reach movements to objects in different directions and depths. However, how networks of cortical neurons compute reach depth from binocular visual inputs remains largely unknown. To bridge the gap between behavior and neurophysiology, we trained a feed-forward artificial neural network to uncover potential mechanisms that might underlie the 3D transformation of reach depth. Our physiologically-inspired 4-layer network receives distributed 3D visual inputs (1^st^ layer) along with eye, head and vergence signals. The desired motor plan was coded in a population (3^rd^ layer) that we read out (4^th^ layer) using an optimal linear estimator. After training, our network was able to reproduce all known single-unit recording evidence on depth coding in the parietal cortex. Network analyses predict the presence of eye/head and vergence changes of depth tuning, pointing towards a gain-modulation mechanism of depth transformation. In addition, reach depth was computed directly from eye-centered (relative) visual distances, without explicit absolute depth coding. We suggest that these effects should be observable in parietal and pre-motor areas.

## Introduction

Aiming to an object in three-dimensional (3D) space requires the transformation of the early visual representation of hand and target position into a desired movement vector specified relative to the effector, i.e. the arm [1], [2], [3], [4]. This visuomotor transformation has been well characterized experimentally in the azimuth/elevation dimensions [1], [5], [6], [7], [8], [9] and the theoretical processes involved [1], [10], [11], [12], [13], [14], [15], [16] as well as the underlying neurophysiology [2], [17], [18], [19] is fairly well understood. However, Blohm and Crawford [1] have recently shown that the distance (i.e. radial depth) of the hand and the target is a major component that has to be taken into account in the visuomotor transformation process. Much less is known about how and where depth information is transformed from early visual coordinates into effector-centered coordinates in the brain [20]. In particular, no model predictions exist regarding what neural properties electrophysiologists might expect to find at the tip of their electrodes when recording from depth transformation areas [20], [21], [22], [23], [24], [25], [26].

In contrast, the encoding of object distance in the early visual system is relatively well understood [27], [28], [29], [30], [31], [32], [33], [34], [35], [36], [37]; retinal disparity selective neurons have been found in many areas of the striate and extra-striate cortex and provide visual distance information relative to fixation distance, i.e. relative distance as opposed to the absolute distance of the object relative to the body. This information is transmitted to the posterior parietal cortex (PPC), an area believed to be involved in the visuomotor transformation for reaching [2], [17], [18], [19]. Pioneering electophysiological studies recording from the PPC have found that in addition to azimuth/elevation receptive fields, neurons are also modulated by fixation distance (vergence) as well as target/hand depth, pointing towards a code of 3D position [20], [21], [22], [23], [24], [25], [26]. In addition, disparity fields in PPC have been reported to at least partially shift with vergence angle [23], [26]; since a perfectly shifting disparity field represents the summation of relative distance and fixation distance to generate absolute distance, these results are an indication of the PPC contribution to the relative-to-absolute distance transformation.

On the motor planning side, it is known that neurons in the pre-motor cortex have 3D tuning fields corresponding to all 3 spatial dimensions, whether encoded explicitly in spatial coordinates or implicitly in a muscle-related reference frame [8], [38], [39], [40], [41]. In other words, a neuron in these areas is preferentially active for movements in a certain direction in 3D space and its activity drops as the angle of the movement from the preferred direction increases.

The lack of theoretical investigations has prevented neuroscientists from searching for signals specifically related to the visuomotor transformation of depth. The only theoretical study investigating depth coding focused on distance representations [42]. However, one finding from the study is relevant to the visuomotor transformation of reach depth, i.e. vergence-related gain modulation. Gain modulation of a neuron does not change its receptive field location or shape but up- or down-regulates its overall activation, typically as a function of body geometry signals such as eye or head orientations. It is known to contribute to motor planning, sensory-motor transformations and multi-sensory integration [14], [15], [16], [43], [44]. Within a population of neurons, gain modulation can alter the relative contribution of each neuron to a certain computation resulting in very different overall population outputs [44]. The goal of the present paper is to provide the theoretical foundations of how distance (i.e. motor depth) can be accurately computed in effector-centered coordinates through distributed processing from distance encoded in a visual reference frame. Moreover, our aim was to provide testable predictions as to what properties neurons involved in this process might display.

To this end, we trained a physiologically inspired artificial feed-forward 4-layer neural network to perform the 3D reference frame transformation for reaching. To train this network, we used a 3D geometrical model describing the analytical relationship between sensory inputs and the ideal 3D reach [1]. The properties of this network with respect to the more classical approach of angular direction information have been analyzed elsewhere [13]. Here, we will specifically focus on the depth-related processes of this 3D visuomotor transformation for reaching. We analyzed the modulation of the network units' receptive fields with distance-related signals (such as vergence, hand distance and target distance) and show that our model reproduces PPC neuron properties. Based on this analysis, we make specific testable predictions as to how fixation depth, reach distance, retinal disparity as well as horizontal eye orientation might influence depth-related neuronal activity in areas such as the parietal and pre-motor cortices, known to be involved in the reference frame transformation for reaching [2], [18], [19]. Finally, we propose a potential general mechanism by which relative distances could be directly transformed into motor depth without requiring explicit absolute depth codes.

## Results

**Figure 1:**
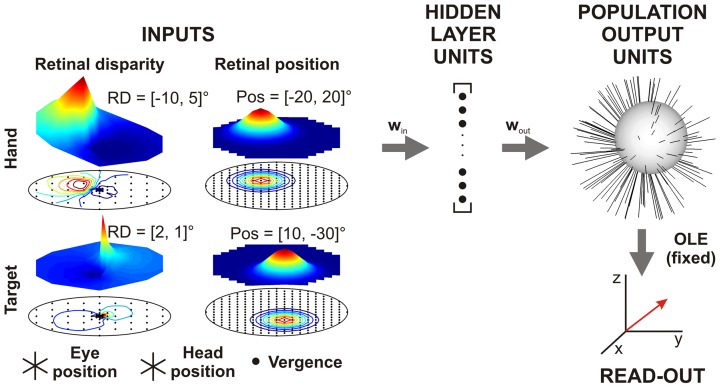
Neural network model. Network inputs consisted of retinal disparity maps (67 units each) for hand and target positions, retinal position maps (253 units each) for hand and target positions as well as 3D eye and head orientations signals (6 push-pull units each) and a vergence input (1 unit). Example population activations (color surfaces above maps of units) are shown for two different retinal disparities and retinal positions (hand and target). All inputs were fully connected to the 2^nd^ (hidden) layer composed of 200 units through weight matrix w_in_. All hidden layer units (HLUs) were fully connected to the 125 population output units (POUs) through weight matrix w_out_. To train the network, we designed an optimal linear estimator (OLE, weights fixed) read-out layer in which each of the 3 units represented one dimension of the decoded motor vector. See text for more details.

The goal of this study was to propose potential physiological properties of neurons that are involved in the 3D visuomotor transformation of depth for reaching. Therefore, we built a 4-layer artificial neural network (Figure 1) designed to mimic the macroscopic anatomy of brain areas mediating this reference frame conversion and trained it to perform the full 3D visuomotor transformation for reaching (see Methods for more details). After ensuring good network performance, we analyze the emerging properties of the network, specifically focusing on the visuomotor transformation of reach depth.

After 10,000 training steps, the network performance reached a mean (±SD) absolute movement error of 4.56±3.42cm. Another performance requirement for the network was to ensure that the network did indeed use extra-retinal signals in the visuomotor transformation. To quantify this, we computed the 3D compensation index (see Methods) as the slope of the overall observed 3D compensation (i.e. network performance) relative to the predicted compensation and found a compensation index of 0.985. This means that the network almost perfectly accounted for the 3D geometry to generate a motor plan. We also specifically computed the depth-related motor error as being 2.21±2.17cm and found that the depth-related 3D compensation index was 0.984. These values were better than the ones typically found in human experiments [1]. After ensuring the good performance of the network, we can now begin analyzing the emerging HLU (2^nd^ layer) and POU (3^rd^ layer) properties.

**Figure 2:**
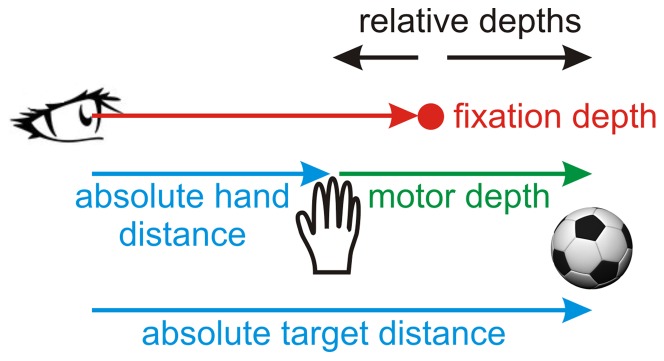
Reaching depth codes. Schematic showing the relationship between relative distance, absolute distance, fixation distance and movement depth.

Before diving into the analysis of the network, let us first have a closer look at the problem that needs to be solved during the depth transformation. Figure 2 depicts the different depth signals that we will consider. The retinal distance information that the brain receives about initial hand position and the target location is a relative depth signal, i.e. relative to fixation depth. The brain has information about fixation depth through the ocular vergence angle (and other retinal cues, but we will only consider conditions in complete darkness where those cues are absent). Combining relative distance and vergence, a network can recover absolute distance, if needed [42]. However, for reach planning the brain ultimately only requires motor depth, which can either be computed by subtracting the relative or the absolute hand and target depths. (Note that a reach plan still needs to be transformed into a set of muscle activations using an inverse model of the arm, which might require additional information, e.g. about current joint angles [45], but this was beyond the scope of our model). If this operation were carried out using a gain-like mechanism [3], [13], [14], [15], [42], [44], then we would predict that depth-related activity should be up- and down-regulated in the hidden layer (HLUs) of our network (gain modulation) and as a result, receptive fields should shift in the population output layer (POUs). To gain insight into this mechanism, we will first analyze how depth-related signals modulate azimuth-elevation receptive fields and then how those signals modulate disparity fields.

### Visual receptive field modulations with depth

**Figure 3:**
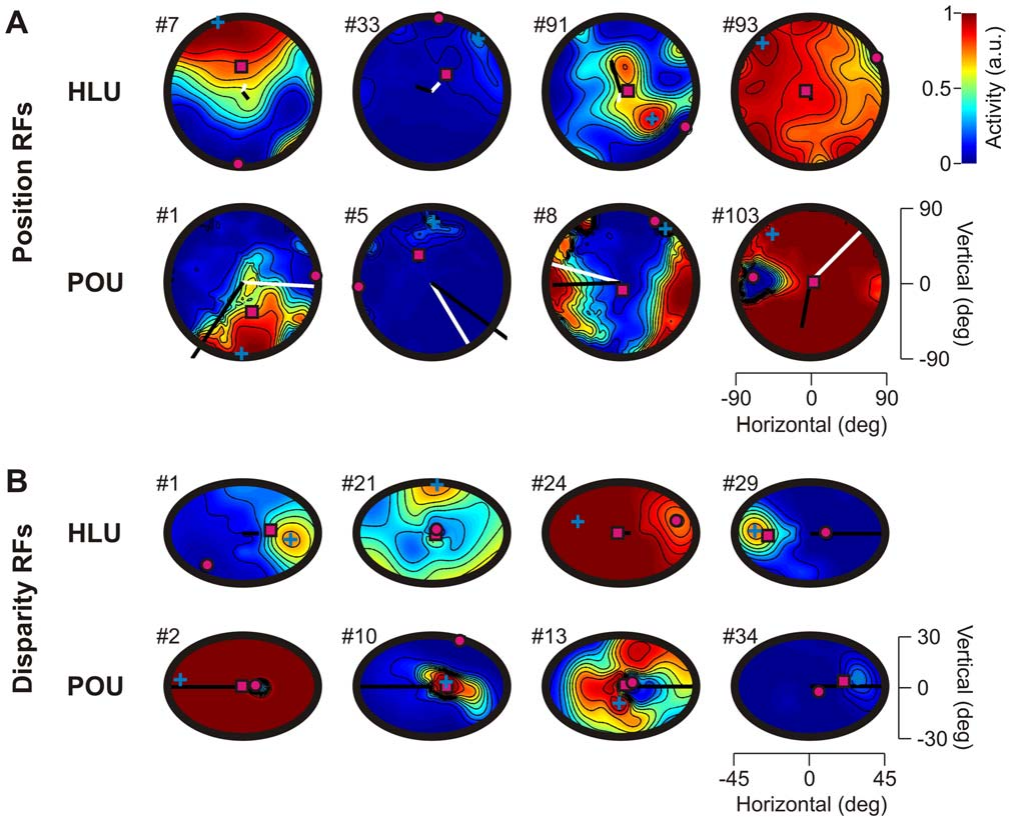
Typical retinal position and disparity receptive fields and depth gain modulation. **A**. Position receptive fields have 90deg limits. Black and white bars indicate eye and head movement sensitivity vectors respectively. HLU: hidden layer units. POU: population output units. **B**. Retinal disparity fields have 30deg horizontal and 15deg vertical limits. Back bars indicate vergence sensitivity vectors. Each receptive field and retinal disparity field is characterized by a maximum (blue cross), minimum (magenta circle) and center of mass (magenta square) of activity. Different preferred distance codings of each HLU are analogous to Gnadt & Mays [21].

Figure 3A shows typical visual position receptive fields (RFs) from HLUs and POUs (90deg visual fields). The RFs were computed while keeping all other input signals constant, i.e. zero eye/head/vergence angles and constant hand/target distances at 50cm. The locations of the RFs' center of mass (magenta square), maximum (cyan cross) and minimum (magenta dot) activation are also indicated. As can be observed, individual RFs can be broadly or narrowly tuned, cover different portions of space and can present different levels of overall activation. The potential eye/head gain modulation on each unit can be seen through the length and direction of the black (eye) and white (head) sensitivity vectors. Eye and head orientation sensitivity vectors represent the direction of eye/head orientation change that maximally affected the position receptive field. Sensitivity vectors are determined by the weight matrices connecting the eye/head input to a unit of interest (see Methods).

In this section, we will analyze how distance-related sensory inputs (fixation distance, i.e. vergence; hand/target distance) modulate RFs of the HLUs and POUs.

#### Vergence-induced RF shifts

**Figure 4:**
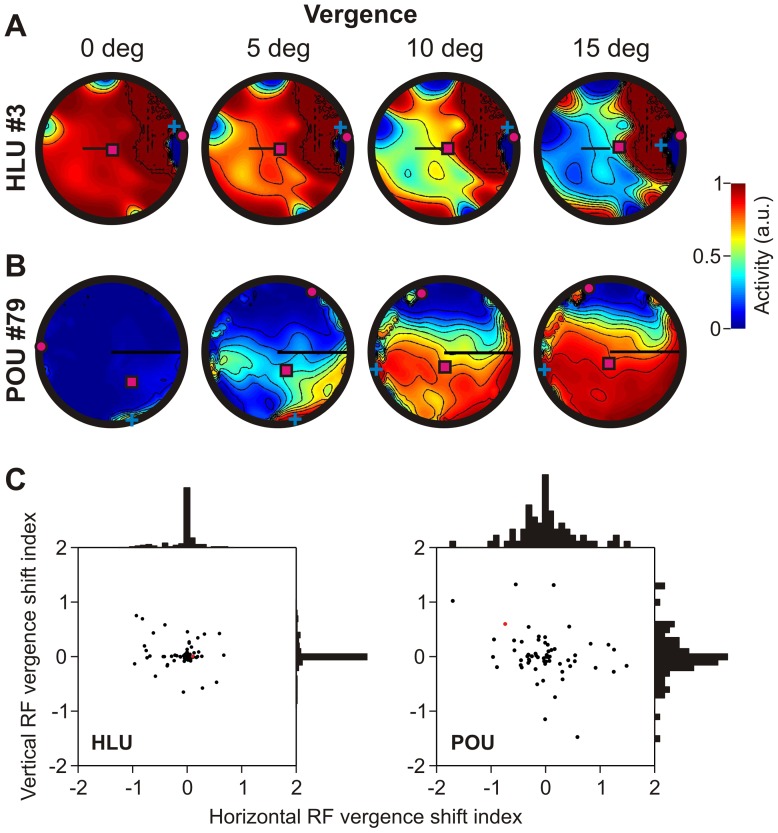
Vergence modulation of visual (position) receptive fields. **A**. Typical example of vergence modulation of a receptive field for a HLU. Black bars indicate the strength of vergence sensitivity. Otherwise the same conventions as in Figure 2 apply. **B**. Typical example of vergence modulation of a receptive field for a POU. Whereas HLUs are only gain modulated by vergence, POU receptive fields tend to also shift with vergence changes, as can be observed by the shift in the center of mass (magenta square). **C**. Indices of horizontal and vertical receptive field shifts due to vergence for HLUs (left) and POUS (right). Normalized histograms show proportion of data points in bin of size 0.1. Most HLUs do not have shifting receptive fields (histogram narrowly centered around 0). Indices of vergence-induced receptive field shifts for POU units show a wide distribution. Red dots indicate examples shown in panels A and B.

First, we were interested in how fixation distance changed the visual (position) RF of HLUs and POUs. This analysis is shown in Figure 4. Figure 4A shows a typical example of how vergence modulates the visual RF of a HLU; Figure 4B shows the same analysis for a POU. As can be observed, the HLU is mainly gain-modulated by vergence; the overall activity (see color legend) varies with vergence angle, but the RF location (indicated by the centre of mass, magenta square) does not change across different vergence angles. In contrast, the RF of the typical POU shifts with vergence, in addition to some modulation of the overall activity.

We quantified the vergence-induced RF shift in Figure 4C. To do so, we computed the horizontal and vertical RF centre of mass for different vergence angles and performed a correlation analysis to determine the vergence-related RF shift index (see Methods section). Figure 4C (left) shows the overall vergence-related spatial shift (horizontal and vertical) in RFs for all HLUs (red dot indicates the example unit shown in panel A). As can be observed, the index was close to zero for almost all HLUs indicating that HLUs generally do not shift their RF for different vergence angles. In contrast, POUs show a wide distribution of RF shift indices, both horizontally and vertically (Figure 4C, right), that was significantly wider than the distribution for HLUs (F-test, p<0.01). Thus, POUs show shifting RFs for different vergence angles (red dot corresponds to example in panel B).

#### Hand/ target depth-induced RF shifts

**Figure 5:**
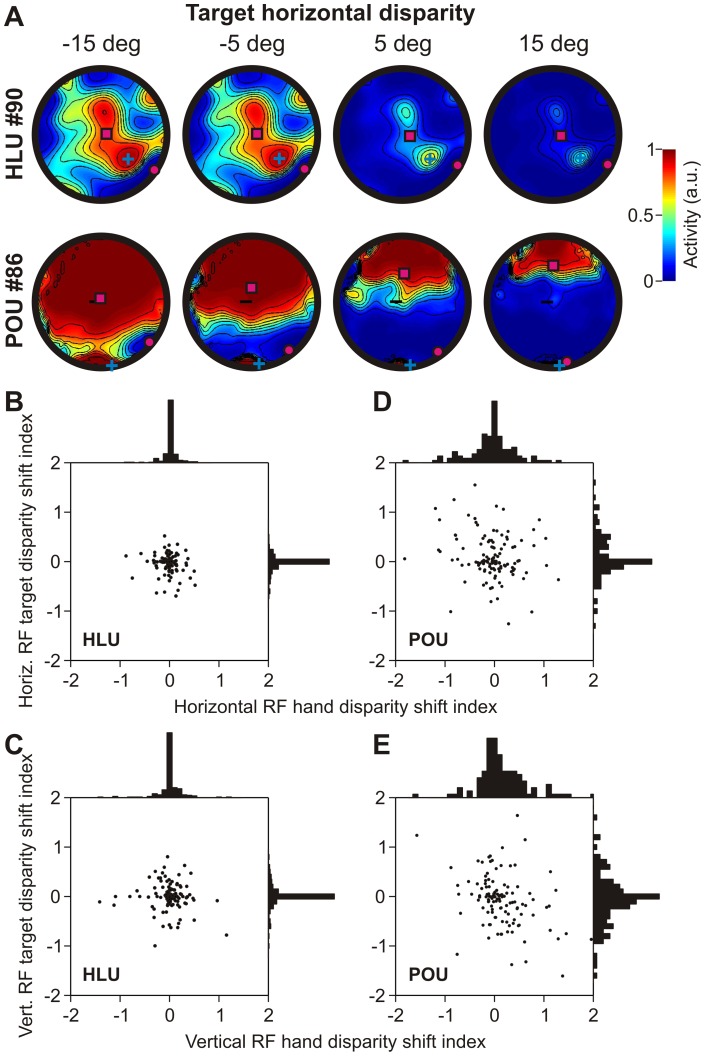
Receptive field modulations with hand/target disparity. Same conventions as in Figure 3, but now showing hand/target disparity-related effects. **A**. Receptive field modulations with horizontal target disparity for a typical HLU and POU unit. **B**. Relationship between horizontal hand and target disparity-induced receptive field shift indices for HLUs. **C**. Same relationship for vertical shift indices. **D**. Relationship between horizontal hand and target disparity-induced receptive field shift indices for POUs. **E**. Same relationship for vertical shift indices shows that POU receptive fields are broadly shifting with changes in hand/target disparity (depth).

Next we analyzed how hand and target depth modulates HLUs and POUs. For example, Figure 5A shows how horizontal target disparity (i.e. target distance relative to fixation distance) modulates the RFs of a typical HLU and POU. Similar to vergence modulation, target depth mainly gain-modulates the HLU activity while the RF location does not shift. This is similar to neural recording results from parietal area LIP, as observed by Gnadt and colleagues [21], [22]. The typical POU, however, shows large RF shifts across different target depths. RF shifts for POUs were significantly larger than RF shifts for HLUs (F-test, p<0.01). This behavior was analogous for changes in hand distance (not shown).

We analyzed how hand and target distance shifted the HLUs and POUs RFs horizontally (Figure 5B and D) and vertically (Figure 5C and E). We did so by computing the RF shift from a regression between RF centre of mass and hand/target disparity. One would expect a negative correlation between hand and target RF shifts if individual units were modulated by the movement vector ( = target – hand) and no correlation whatsoever if the hand and target were modulating RFs independently. Both hand and target disparity induced small or no RF shifts in HLUs, neither horizontally (Figure 5B) nor vertically (Figure 5C). In contrast, the RF of POUs showed a wide distribution of shifts in both horizontal (Figure 5D) and vertical (Figure 5E) directions, significantly wider than in HLUs (F-test, p<0.01). We did however not find any significant correlation between hand and target RF shifts in either of the directions or layers (p>0.24 for both horizontal and vertical indices across HLUs and POUs), suggesting that hand and target information is coded independently. This does, however, not preclude the possibility that the motor vector is transformed at a population level.

#### Gain modulation

**Figure 6:**
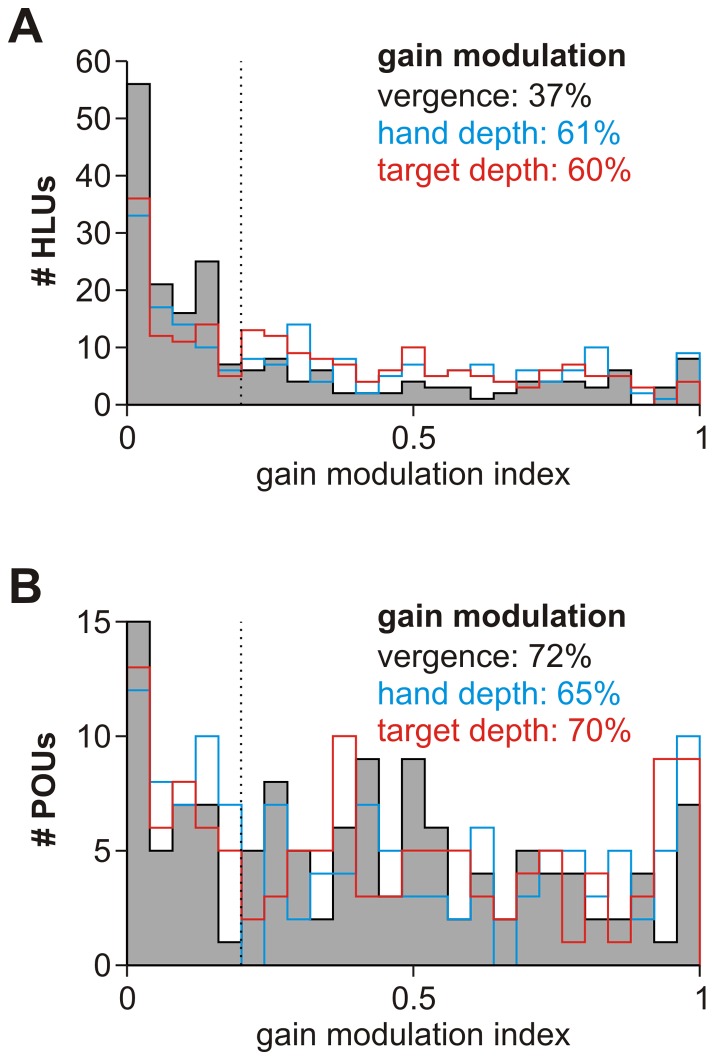
Vergence, hand and target depth gain modulation. **A**. Summary of gain modulation analysis for HLUs. Vergence (black), hand depth (cyan) and target depth (red) gain modulation indices are shown as a histogram for all HLUs. We used an arbitrary threshold of 0.2 to determine the percentage of “significantly” modulated units. Note that vergence modulated HLUs less than hand/target depth. See Methods section for calculation of the gain modulation index. **B**. Gain modulation summary for POUs. Vergence, hand and target depth had similar effects.

To quantify how vergence, hand and target depths gain modulate the strength of unit activity, we computed gain modulation indices similar to Bhattacharyya et al. [26] (see Methods section). The results of this analysis are shown in Figure 6A for HLUs and Figure 6B for POUs. While there was a wide range of gain modulation values, it is worth noting that – similar to previous experimental findings [26] – vergence modulated significantly fewer HLUs than hand/target depth, while this was not the case in POUs (see Discussion Section).

### Retinal disparity fields and depth coding

**Figure 7:**
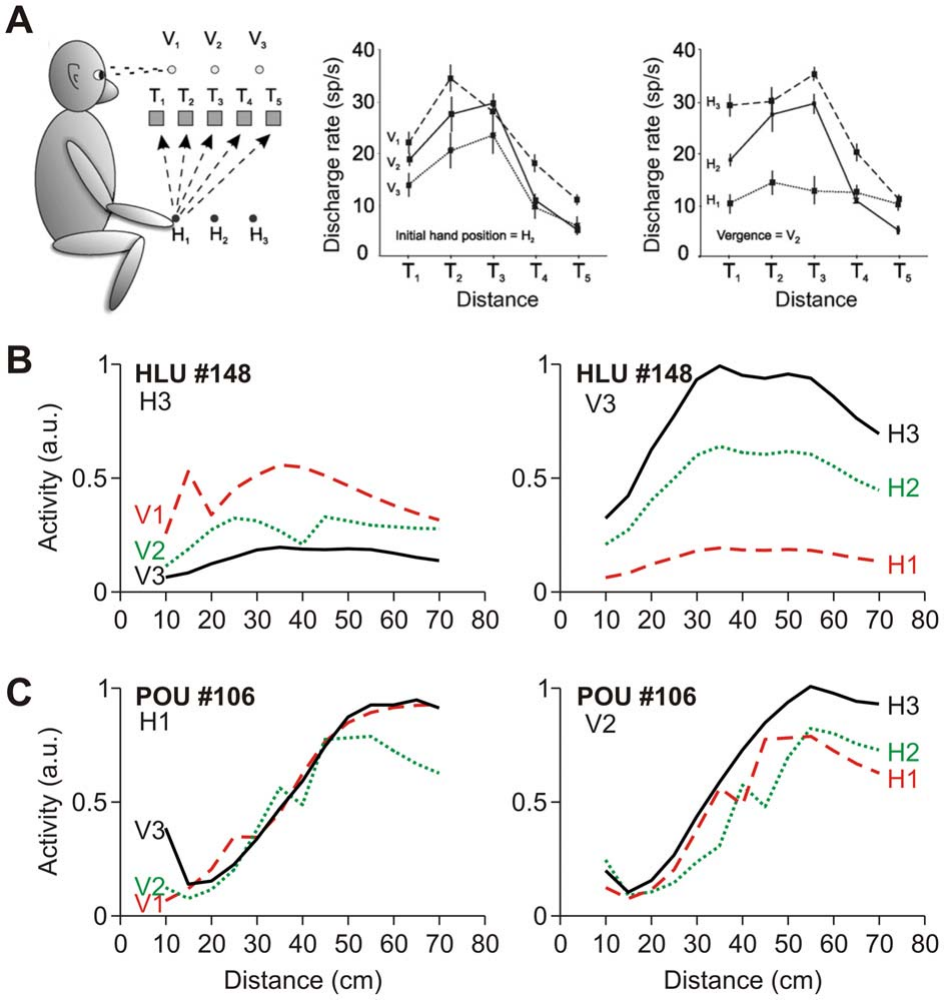
Simulation of experiments performed by Ferraina et al. [20]. **A**. Original data from Ferraina et al. [20]. Left panel shows a schematic of the setup with 5 different target positions (T), 3 different initial hand positions (H) and 3 different fixation distances (vergences, V). Center and right panels show the modulation of neuronal activity across target distance with vergence and initial hand position distance respectively. **B**. Typical hidden layer unit activity under similar simulated conditions. Same representation as in panel A. **C**. Typical population output unit activation under the same conditions. Note that while HLUs were strongly modulated both by vergence and hand distance, POUs were generally not modulated by vergence but only by initial hand position.

We have seen in the previous section that hand, target and fixation distance *independently* modulate the receptive field of HLUs and POUs. Next, we will analyze how activity changes with combined hand, target and fixation distance modulations. This analysis was directly inspired by Ferraina et al. [20], who recorded neuronal activity during such a task in area PE within the PPC. Their main results are reproduced with permission in Figure 7A. Monkeys were asked to reach from different hand distances to targets at different distances while fixating in different depths (Figure 7A, left). For this typical neuron, discharge as a function of target distance was essentially gain modulated by both initial hand distance (Figure 7A, right) and fixation distance (Figure 7A, center).

We simulated the exact same experimental set-up as Ferraina et al. [20] in our network. Figure 7B shows the result of a typical HLU plotted in the same way as in Figure 7A. As can be observed, the HLU nicely reproduced the main findings of Ferraina et al. [20], i.e. showing gain modulation with both fixation distance (vergence, V1-3, Figure 7B, left panel) and initial hand distance (H1-3, Figure 7B, right panel). This was the case across the majority of HLUs. In contrast, the observed network behavior was quite different in POUs. Figure 7C shows a typical POU whose activity remained largely unaffected by hand and fixation distance. Again, this was true for the majority of POUs.

These results are a first indication that POUs code more for movement-related parameters (absolute movement depth) than for visual parameters (relative distance). Next, we will elaborate on this observation and perform a more detailed analysis of how distance is encoded and transformed in our network.

#### Vergence modulation

In order to investigate how depth is coded in our network, we began by looking at how target retinal disparity (RD) fields were modulated by vergence, i.e. fixation distance. Examples of typical RD fields are shown in Figure 3B. A RD field is a unit's response to different combinations of horizontal and vertical retinal disparities arising from different target distances (horizontal axis: left is further, right is closer) and binocular torsion values (up corresponds to clockwise torsion) respectively [46]. For example, HLU#29 prefers stimuli that are located further away than fixation distance, i.e. negative disparity tuning (e.g. Figure 3B). For disparity fields, sensitivity vectors show the strength and sign of vergence modulation. We hypothesize that if RD coding does not change with vergence, then a unit is coding for relative distance (i.e. relative to fixation distance). In contrast, modulations with changes in vergence would be indicative of a transformation between relative depth and absolute depth or movement distance.

**Figure 8:**
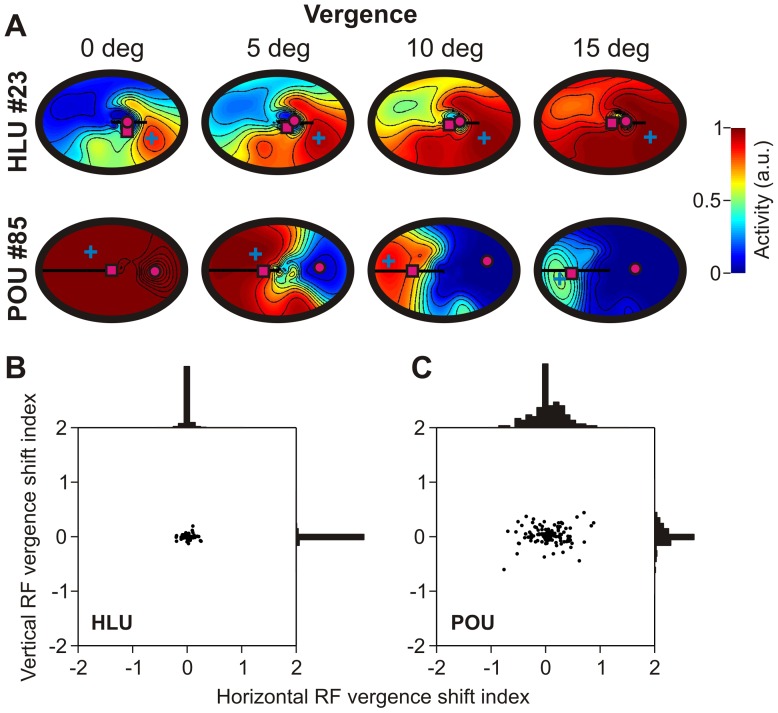
Vergence modulation of retinal disparity receptive fields. **A**. Typical HLU and POU unit activity modulation with ocular vergence angle. Same representation as in Figure 2. **B**. Horizontal and vertical receptive field shift index with vergence angle for all HLUs. Histograms show that shift indices center tightly around zero. Vergence-related activity modulation in HLUs is mainly due to gain-like mechanisms. This is analogous to Genovesio & Ferraina [23]. **C**. Same receptive field shift indices for all POU units displays a much broader distribution of indices.

Figure 8A shows a typical HLU and a typical POU retinal disparity field across different vergence angles. While the HLU RD was gain modulated by vergence, it did not shift preferred depth (magenta square, centre of mass). In contrast, the typical POU showed a shift in preferred RD coding with vergence. To analyze these observations more quantitatively across the entire network, we performed a regression analysis similar to the one carried out for receptive fields in the previous section. We correlated RD preferred depth shifts with vergence changes and plotted the results in Figure 8B (HLUs) and Figure 8C (POU). This analysis confirmed our observation from the typical network units in Figure 8A. HLUs were mostly gain-modulated (not shown) but did not show significant horizontal or vertical preferred RD shifts (Figure 8B). However, POUs showed large RD shifts for horizontal disparity (Figure 8C), significantly larger than HLUs (F-test, p<0.01). This is in line with the finding that horizontal disparity is mainly responsible for distance coding, while the role of vertical disparity is less clear [47]. These observations are analogous to and consistent with findings from Genovesio and Ferraina [23] of neurons in the lateral intraparietal areas (LIP). More importantly, Bhattacharyya et al. [26] show that reach-related neurons in the parietal reach region of PPC display properties that are almost identical to our HLUs. Overall, this analysis shows that while HLUs mainly code for relative distance, POUs seem to shift their coding more towards absolute distance or movement distance.

#### Combined depth

**Figure 9:**
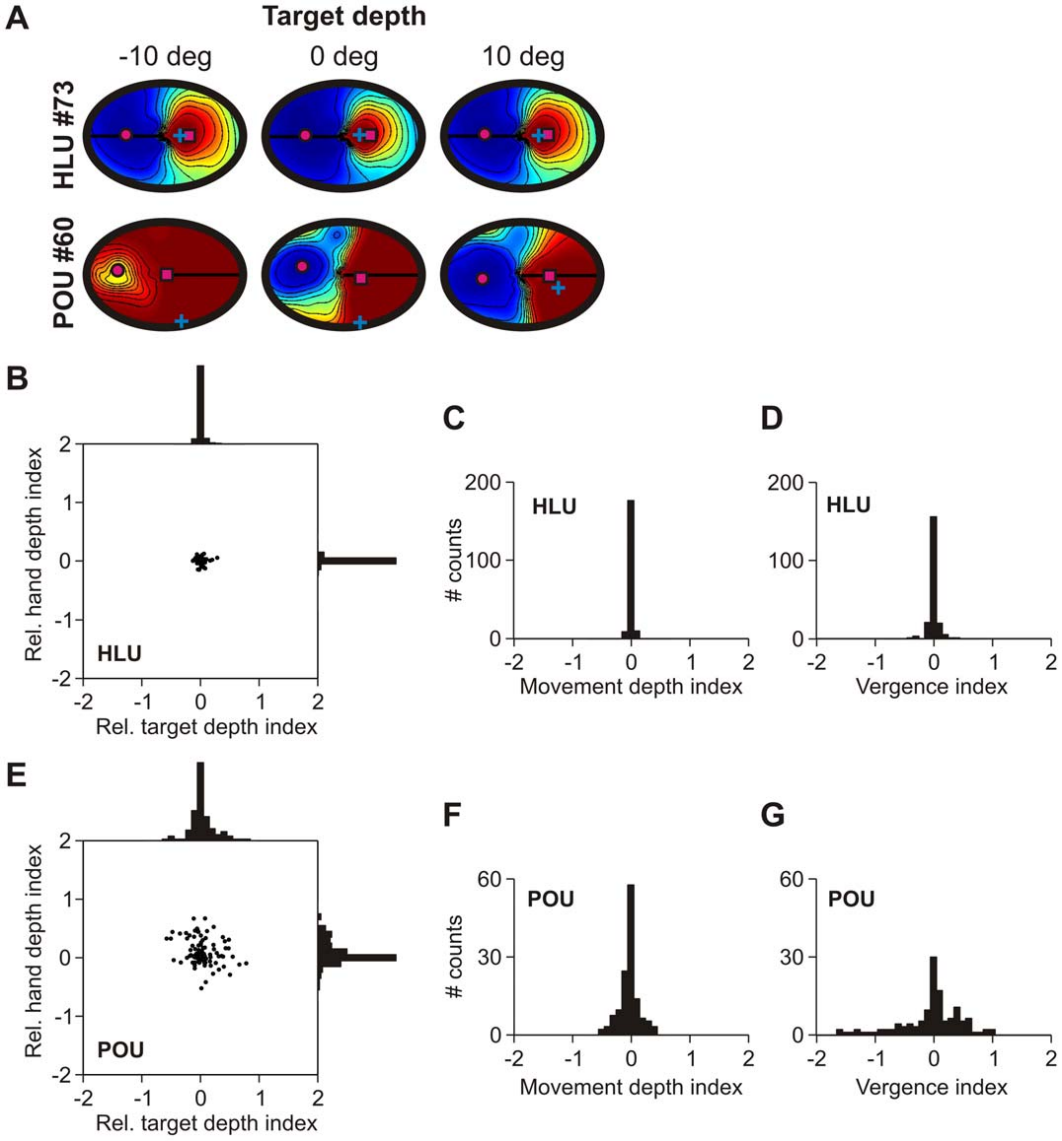
Relative versus absolute distance coding. **A**. Changes of typical hand RD fields with target depth (coded in degrees of disparity). The HLU shows some gain modulation but no RD field shift, while the POU's RD field shifts with target position, as evidenced by the shifting centre of mass (magenta square). **B–G**. RD shift indices of relative hand/target depth (panels B and E), movement depth (panels C and F) and vergence (panels D and G) for HLUs (panels B–D) and POUs (panels E-G). This confirms the observation from the typical trials in panel A. RD fields of HLUs do not shift, while large shifts are observed for POUs.

To gain further insight into how depth is coded and transformed in the network, we analyzed the combined effects of hand distance, target distance, vergence, and movement distance on the activity of the network units. How these variables relate to each other is depicted in Figure 2. Figure 9A shows the modulation of hand RD for a typical HLU and a typical POU with target depth relative to fixation (i.e. degrees of horizontal disparity). Since both hand and target inputs are visual signals, both hand and target depths are coded through RD fields. Similar to the vergence modulations of RD, HLUs only show gain effects of target distance but no RD shifts, in contrast to POUs who show large preferred RD shifts. Note that we observed qualitatively the same behavior for hand depth modulations of target RD (not shown).

Figure 9B–G shows the RD shift indices with hand, target, fixation and movement distance for HLUs (Figure 9B–D) and POUs (Figure 9E–G). Note that target depth indices were computed on the hand RD fields while all other index calculations were carried out on the target RD fields. Overall, HLUs' RD fields did not (or only slightly) shift with hand or target depth (Figure 9B) but were only gain modulated by these variables (not shown). In contrast, POUs showed significantly larger RD field shifts (F-test, p<0.01) across hand and target depth (Figure 9E) on top of some gain modulation (not shown). We also tested for correlations between hand and target shift indices. A perfect negative correlation would mean that depth coding shifts in equal but opposite directions for hand and target depth, which would be indicative of an invariant movement depth code (since movement depth  =  target depth – hand depth). While we did not find any consistent correlation between the hand and target shift indices in HLUs (R^2^ = 0.003, p = 0.47), there was a significant (although weak) correlation for POUs (R^2^ = 0.05, p = 0.01). We also analyzed how movement depth and vergence modulated RD fields. HLUs show virtually no shifts in RD fields with movement (Figure 9C) and fixation (Figure 9D) depth. In contrast, POUs show a wide distribution of RD shift indices with movement depth (Figure 9F) and vergence (Figure 9G). This points towards a depth code for POUs that is not anymore relative to fixation distance but rather shifted towards movement depth. Thus, to summarize the findings, it appears that while HLUs code relative distance (which is consistent with [26]), POUs might code absolute distance or movement distance in a distributed way.

#### Depth transformation

**Figure 10:**
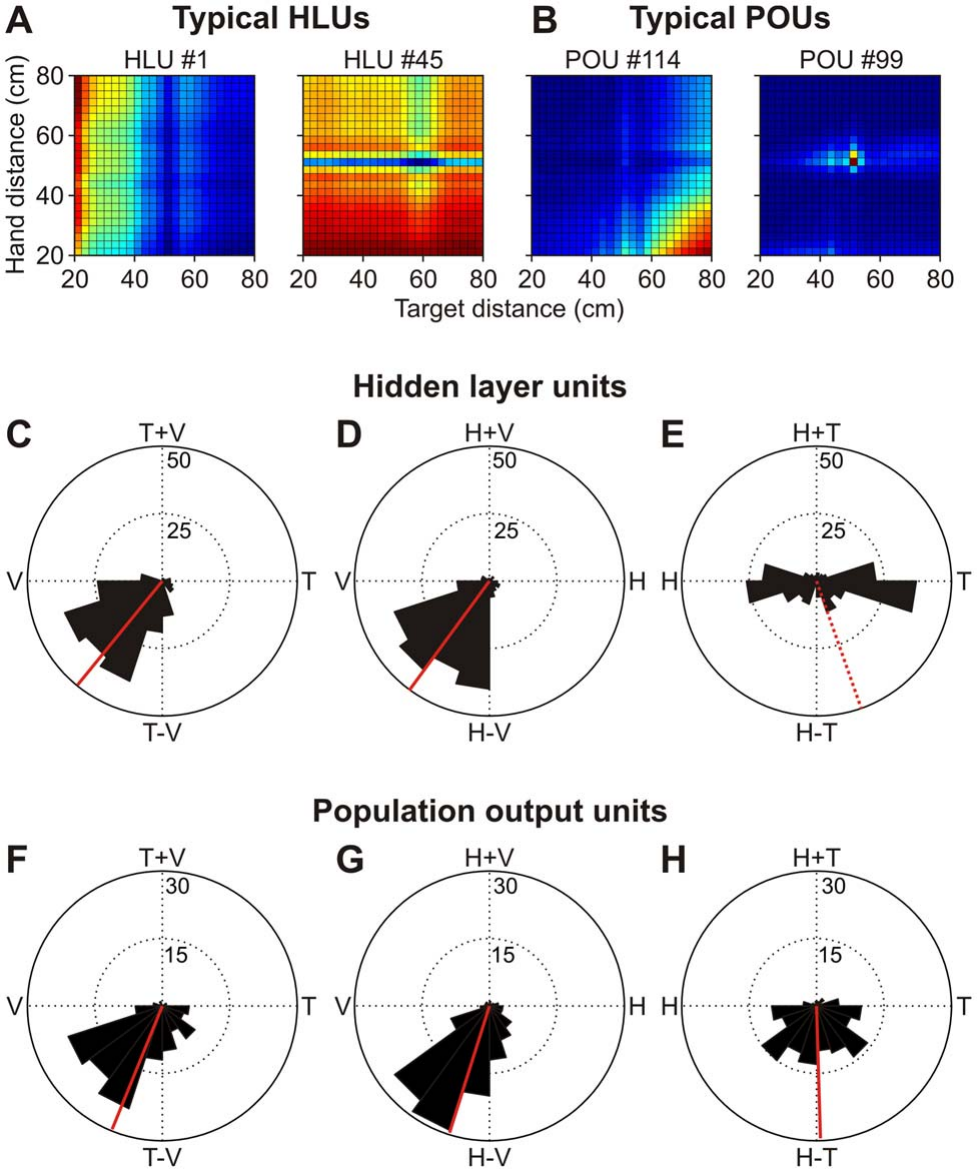
Depth separability index. **A**. Hand-target depth modulation for two typical HLUs. Color coding shows unit activity levels (same color scale as in Figure 7) for different combinations of hand and target distance. **B**. Hand-target depth modulation for two typical POUs. HLUs' activity is modulated either in the hand or in the target direction but not both, while POUs' activity shows maximal activity for a specific combination of hand-target distance. **C–H**. Separability plots for target-vergence depth dependencies (panels C and F), hand-vergence depth dependencies (panels D and G) and hand-target depth dependencies (panels E and H). HLUs code hand and target distance separately (panel E); POUs mainly code for the difference between hand and target distance (panel H).

Our final analysis addresses whether hand, fixation and target distance are coded jointly or independently. To do so, we computed the depth separability indices, similar to what has previously been done for movement coding in the fronto-parallel plane [13], [48]. To do so, we first computed the units' activity pattern for different combinations of hand, vergence and target distance, while keeping all other inputs constant. Examples of these activity patterns for hand-target distance changes can be seen in Figure 10A for two typical HLUs and two typical POUs. Note that typical HLUs are mainly modulated either along the hand or target distance axis, but not both, while typical POUs display combined hand-target codes. This means that these two HLUs seem to code hand or target direction separately, while the two POUs seem to code for a combination of hand-target distance.

To quantify this observation across all units, we computed the separability index for hand-vergence-target combinations by averaging the local response field gradient directions of the depth modulation patterns (e.g. Figure 10A and B) and multiplied it by 2 modulo 2π [13], [48]. If the response field gradient is mainly horizontal or vertical (i.e. the unit activity is modulated by one variable only, as for HLUs, Figure 9A), then the result of this computation is either 0deg or 180deg. Combined modulation of response fields (such as for POUs, Figure 10B) will result in −90deg (subtractive interaction) or +90deg (additive interaction of variables). Thus each unit is characterized by one angular separability value. The results of this computation are plotted as polar histograms in Figure 10C–E (HLUs) and Figure 10F–H (POUs) for target-vergence distance combinations (panels C and F), hand-vergence distance combinations (panels D and G) and hand-target distance combinations (panels E and H). As can be observed, hand/target and vergence are generally coded – at least partially – in conjunction for both HLUs (Figure 10C, D) and POUs (Figure 10F, G). This is apparent from the fact that most units have separability indices that lie in between V and T–V (panels C and F) or that lie in between V and H–V (panels D and G), which is similar to what has been found in parietal cortex [26]. However, hand and target distance are coded independently in HLUs (Figure 10E), indicating that movement depth has not been computed yet at this stage of the processing. However, the difference between hand and target is generally coded in the POUs (Figure 10H); this difference is equal to the desired movement depth. These results point towards a transformation of relative and independent hand/target distances into an absolute movement depth.

### Depth from eye/head rotations

**Figure 11:**
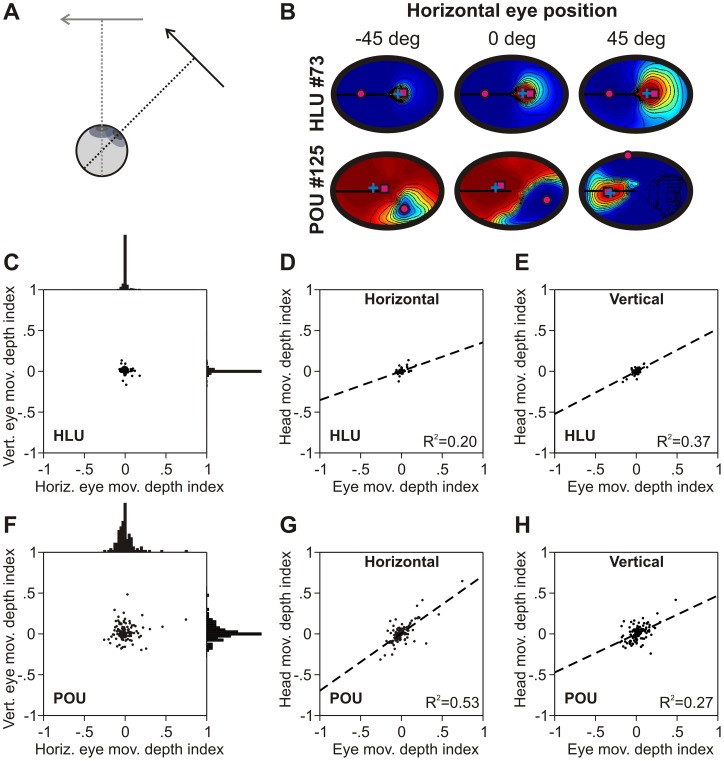
Depth from eye/head rotations. **A**. Schematic illustrating how eye/head rotations of the same visual hand-target vector lead to different motor depths. **B**. Modulation of RD fields with horizontal eye position for typical HLU and typical POU. The HLU is gain-modulated by eye position, but it's preferred RD does not shift. The preferred RD of the typical POU shifts significantly (magenta square). **C, F**. Indices of RD shift due to horizontal and vertical eye position changes for HLUs (no shifts, panel C) and POUs (wide distribution of shifts, panel F). **D, E, G, H**. RD shift indices for horizontal (panels D, G) and vertical (panels E, H) eye versus head rotations for HLUs (panels D, E) and POUS (panels G, H). There was a significant correlation between the eye and head indices both horizontally and vertically throughout the network.

Retinal inputs depend on the eye-head orientations. As a consequence, the same retinal input is interpreted differently by the motor system for different eye-head orientations (see Figure 11A). Therefore, the same retinal movement vector should result into different movement depths depending on the eye-head orientation. While the consequences of this are known for the horizontal-vertical plane [1], [2], [17], [19], this has not been explicitly considered with respect to the visuo-motor transformation for depth. Here we ask whether the RD fields are also modulated by eye-head orientation, which would be required to take this aspect of the visuomotor transformation into account.

Figure 11B shows the RD for a typical HLU and a typical POU and for different horizontal eye orientations. Clearly, eye position affects both typical units' RD field. The HLU's RD field is gain modulated by eye position, while the POU's RD preferred location also shifts in addition to gain modulation. We again performed a regression analysis of RD field shifts as a function of horizontal and vertical eye movements. Figure 11C and F show the results for HLUs and POUs respectively. This analysis confirms that HLUs do not generally shift their preferred RDs, but the preferred RDs for POUs show a significantly wider range of shifts (F-test, p<0.01). This indicates that the depth changes due to eye rotations are accounted for by the network.

This effect was also observed for both eye and head rotations. Figure 11D, E shows the relationship between the RD shift indices due to horizontal (panel D) and vertical (panel E) eye and head rotations for HLUs. Although there were almost no RD shifts, there remained a significant correlation between the small eye and head movement depth indices. For POUs, the eye and head movement depth shift indices were much larger (Figure 11G, H) and were also correlated significantly. Since eye and head rotations have the same effect on motor depth (i.e. the eyes rotate with the head when the head moves), this shows that the network accounted for eye-head-rotation induced depth changes in a consistent fashion.

## Discussion

We trained a physiologically inspired feed-forward neural network to perform the 3D visuomotor transformation for reach depth. Emerging properties of our network were consistent with all known electrophysiological findings about distance coding in the cortex, which validates our approach. In addition, we make a number of new predictions as to the receptive field and retinal disparity field properties that one might find when recording in brain areas involved in the visuomotor transformation of reach distance. For example, if the brain uses the same mechanism as our network, then we predict that reach planning areas involved in the transformation of reach depth should show eye/head/vergence-related changes of direction and depth coding and that absolute hand/target distance codes should be rarely observed as a result of a direct transformation from relative distances into movement distance. Finally our network provides a potential mechanistic explanation of how the brain might transform relative distance into movement depth. These points will be discussed below.

### Network comparison to literature

Our network results are in agreement with all electrophysiological data that we are aware of concerning the coding of movement depth in the brain. First we found that HLUs' receptive fields are modulated by retinal disparity (i.e. depth) and have a preferred depth. This is analogous to a 3D position code found in area LIP during saccadic eye movement tasks [21], [22] and the parietal reach region (PRR) in a reaching task [26]. Second, fixation distance (i.e. vergence) gain-modulated HLUs' retinal disparity response while shifting the preferred disparity of POUs. Our HLU properties are in line with recent finding by Bhattacharyya et al. [26] showing that PRR neurons' retinal disparity tuning was gain-modulated by the vergence angle. Area LIP shows intermediate distance codes similar to POUs, as evidenced by a wide distribution of disparity tuning shifts with fixation distance [23]. In addition, gaze direction and ocular vergence angles modulate neural activity in area V6a of the posterior parietal cortex [49] in a way that is similar to HLUs in our network. However, the latter studies were performed using saccadic eye movements and these results differ from brain areas involved in reach planning [26].

To the best of our knowledge, only three studies have investigated the coding of reach depth [20], [24], [26]. They show that posterior parietal cortex (PPC) neurons are tuned for target depth and gain-modulated both by fixation distance (vergence) and initial hand distance (hand disparity). We reproduce these findings in detail (Figure 7) and show that our HLUs are compatible with this PPC code. It is also interesting to note that vergence gain modulation was weaker than hand/target depth gain modulation in HLUs (see Figure 3C, D), which is similar to findings by Ferraina et al. [20], [24]. This was surprising given that our network was not designed to display this effect; rather, this asymmetry was an emerging property of our training. Thus, instead of being an asymmetry between the role of vergence and hand/target distance in the brain, these results suggest that this is simply the optimal way for a distributed network to get the job done. These observations point towards a gain-modulated relative distance code in the parietal cortex that is in eye-centered coordinates [26]. This is also consistent with previous studies that have shown 2D reach coding in eye-centered coordinates in the PPC [50], [51]. Here, we propose that this type of reach coding in the parietal cortex also applies to 3D space.

### Potential mechanisms for depth transformations

In this study, gain modulation emerged through self-organization of a trained network. Presumably, this was because gain modulation is the only known mathematical way that reference frame transformations can be achieved in a feed-forward neural network. Using gain modulation, the network gradually transforms egocentric, relative depth inputs into motor depths, by weighing units' with different preferred depths differently depending on the sensory context, i.e. depending on vergence, eye-head orientations and hand/target depth. Therefore, we interpret the presence of gain modulation across all layers of our network as providing the computational foundations for the visuomotor transformation of depth. Also compatible with this concept is the observation of a wide range of different receptive field and retinal disparity shift indices in the POU of our network as a result of the gain-weighted combination of different HLUs. We therefore suggest that the visuomotor transformation of reach depth might also rely on gain modulation mechanisms as has previously been found for azimuth and elevation [3], [12], [13], [14], [15], [16], [43], [44].

We have shown that motor depth can be calculated directly through distributed computing without having to explicitly compute absolute distance (from relative depth and vergence). As a result, we observe mixed or intermediate depth codes that are neither relative, nor absolute, nor reflecting purely motor depth. Although some network units (POUs) did show absolute distance coding (see Figures 8, 9, 10), Figure 10 suggests that absolute distance is a by-product of transforming relative depth into motor depth in a distributed manner rather than a requirement.

For perception, the conversion of relative distance to egocentric (absolute) depth has been investigated theoretically in the past [42]. Interestingly, our network shows that the intermediate stage of coding depth in absolute terms is not a requirement in the reach system. Instead, relative depth is readily converted into movement depth without transitioning explicitly to absolute depth. Therefore, in contrast to the perceptual system where absolute distance might be required [42], [52], our network predicts that this might not be the case for the sensory-motor transformation underlying reaching.

To carry out the 3D visuomotor transformation of depth, the presence of ocular version and vergence signals in the network is a crucial requirement. Interestingly, eye version effects on the 3D position code have also been observed in neuronal recordings in the parietal cortex [49], [53], which might be indicative of a role of parietal cortex in the depth transformation. Our network shows how eye (and head) position gain fields are crucial for rotation-dependent depth changes and to transform eye-centered motor depth into spatially accurate movement distances.

### Predictions and limitations

Since only few neurophysiological studies on the coding of reach depth in the cortex exist, the main strength of our network is to make a series of testable predictions. Of course, these predictions are based on the specific way that our network solved the problem, which might be different from how the brain does it. However, given the similarities between our network properties and recordings from the real brain, we might nevertheless provide useful predictions. Structurally speaking, we hypothesized that HLUs represent parietal cortex areas and POUs are similar to pre-motor areas in the brain. However, it is also possible that the 3D visuomotor transformation is carried out more gradually across many different areas. In that case, all our predictions would still be valid, but there would be a less clear cut difference between areas showing only gain modulations and areas also showing shifts in their tuning curves; in that case, a more gradual transition between those behaviors would be expected.

One of the strongest predictions of our study concerns the separability of hand, target and fixation distances (Figure 10). While gain modulations of disparity tuning with hand and fixation distance are present [20], [24], [26], we predict that there should also be modulation of target distance and moreover that they should be coded independently in PPC. In agreement with this prediction is the recent finding that vergence and disparity coding is separable in PRR [26]. In addition, the monotonic interaction of vergence with disparity tuning (through gain modulation) should lead to partially inseparable coding of vergence and hand/target distance in PPC (see Figure 10). In contrast, pre-motor areas in the brain (such as PMd) should show inseparable, combined coding of hand and target distance, compatible with a movement distance code.

In general, the coding of a motor plan in depth in pre-motor areas is a wide open question. The implication of pre-motor cortex as a potential functional equivalent to POUs is solely based on previous speculations [13], [38], [41], [44] and remains to be shown. From POUs in our network, we predict that pre-motor receptive fields should shift with hand/target distance (retinal disparity) and with ocular vergence (see Figures 4 and 5). In addition, pre-motor disparity tuning should also shift with vergence and hand distance (Figure 8 and 9), as required to establish a final motor plan in depth. The disparity tuning in pre-motor areas should also shift with eye/head rotations to reflect the depth changes resulting from rotating eye-centered motor plans (Figure 11).

There are many limitations to this network model. For example, in the real brain, sensory information about the hand arises from proprioception in addition to vision. As a consequence, when both signals are simultaneously available, the neural network underlying reach planning must also solve the multi-sensory integration problem [45], [54], [55], [56]. In addition, our network is purely static, but in the real brain these computations are carried out in a dynamic fashion and using spike codes. Also, our rate-based network does not follow cortical architecture, which might influence network performance [57]. For example, the described transformations could be carried out by more than 2 hidden layers, in which case we would expect a more gradual transformation of relative distances into movement depth. There might also be slight differences in emerging network properties depending on the actual training algorithm used, although Blohm et al. [13] have not found any qualitative differences when using other training methods or network sizes. Therefore, those and many other extensions of our current model are possible in future research.

In summary, we have shown that simple feed-forward neural networks can capture in much detail the visuomotor transformation of depth, which expands on previous findings for angular direction [13]. Based on our network, we provide a potential mechanistic explanation for depth transformations in the brain, relying upon gain modulation of depth tuning; 3D spatial tuning curves are gain weighted by binocular eye and head orientation signals to directly produce reach depth from visual inputs and through distributed coding. The main strength of our network approach is that it bridges algebraic (lumped) models, behavior and neurophysiology. As a result, we can attempt to make specific testable predictions of neuronal properties that one might find in areas involved in transforming reach depth from visual to effector-centered coordinates.

## Methods

### Model overview

The visuomotor transformation for reaching in azimuth and in depth can be divided into three consecutive stages. First, the brain must combine binocular 2D retinal images to build and maintain an internal egocentric representation of 3D hand and target positions [51], [58], [59], [60], [61]. Second, these gaze-centered hand and target codes have to be transformed into a 3D motor plan that is specified with respect to the effector [2], [18], [19], [62]. Third, the brain needs to convert this motor plan into appropriate muscle activations to drive that arm to the target [39], [63], [64], [65]. Here, we focus on the second stage of this process and specifically ask how hand and target distances are converted into appropriate movement distances.

Our model consisted in a physiologically inspired, fully connected feed-forward neural network approximating the complete 3D open-loop visuomotor transformation for reach planning [1]. Figure 1 shows a schematic of the model architecture. To address this visual-to-motor transformation process of hand and target distance into reach depth, we used 3D gaze-centered visual population codes and 3D effector-centered movement population coding as the network's inputs and output respectively. The visual input of both hand and target was composed of a 2D retinal angular direction map and a 2D retinal disparity map. Extra-retinal (monocular) eye, head and vergence signals were also required to perform the 3D reference frame transformation [1]. We chose to use only visual initial hand position and no explicit proprioceptive information as an input because it has been shown that in the absence of vision the posterior parietal cortex encodes hand position in gaze-centered coordinates [66]. Studying multi-sensory integration of proprioceptive and visual initial hand positions in a network model should be the main focus of a separate future study. The analysis of a similar neural network [13] with respect to the visuomotor transformation of target elevation and azimuth has produced network properties that were fully compatible with electrophysiological results [41], [43], [48], [50], [66], [67], [68], [69], which validates our approach. All input signals were fed into a first hidden layer, then a second hidden layer that we call the population output. The forth layer consisted of 3 units encoding the desired motor vector in effector-centered coordinates (in Euclidean space) and was a read-out for the activity of the population output (3^rd^) layer. We used this read-out to train the network.

The input-output relationship of all network units in the second and third layer was modeled by a sigmoid function, mimicking the non-linear transfer function of real neurons [70], [71], [72], such that
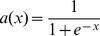
(1)


None of the inputs were subjected to the sigmoid transfer function, nor was the output layer; both were purely linear. Note that we did not use “basis function networks” that force Gaussian (non-monotonic) response tuning, as this has been done in previous studies [14], but instead used monotonically increasing sigmoid transfer functions, as this is physiologically more realistic [70], [71], [72].

### Network inputs

#### Retinal position: topographic hand and target maps

Hand and target azimuth (p_X_) and elevation (p_Z_) angles were encoded in a set of topographically arranged units representing cyclopean retinal positions [73], [74], [75] relative to the fovea. These units had Gaussian receptive fields (width σ = 20deg) and their activations were specified by:
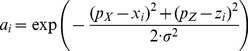
(2)where x_i_ and z_i_ are each unit's preferred directions. Analogous to striate cortex, these units were uniformly distributed on a topographical map with 90deg range. We used a 90deg range despite the fact that inputs were restricted to a 70deg range in order to avoid edge effects. The horizontal/vertical spacing between units was 10deg, which led to 253 units in each retinal position map. Figure 1 shows two example population activations for different retinal positions (hand: [−20deg; 20deg], target: [10deg; −30deg]) as color surfaces over the topographically arranged input units. Similar retinal maps have been used in previous network studies [13], [15], [16], [76].

#### Retinal disparity: topographic hand and target maps

Hand and target horizontal and vertical (d_H_, d_V_) retinal disparities specify the relative distance of the hand/target with respect to the fixation distance (determined by the ocular vergence angle (see below)). Retinal disparity coding neurons had tuning profiles similar to those found in monkey neurons [34], [77] and cats [78], [79], [80]. Here, we used idealized disparity tuning functions that were 2D extensions of previously used ones [42], [81], such that:
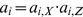
(3)

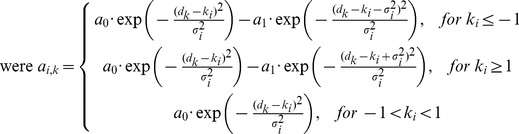
(4)


and where k stands for X or Z, variance 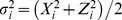 with a minimum variance of 10 minarc and constants a_0_ = 1 and a_1_ = 0.35 [13], [42]. Different combinations (Xi, Zi) of units' preferred disparities were limited to an ellipse (45deg,30deg). Again, the data range was only about (25deg, 10deg) and we chose larger disparity ranges to avoid edge effects. Preferred disparities were spaced at 1deg for disparities <2deg, spaced at 5deg for disparities up to 10deg and were spaced at 10deg anywhere else resulting in a total of 67 units in each retinal disparity map. Figure 1 shows two example population activations for hand ([−10deg; 5deg]) and target ([2deg; 1deg]) retinal disparities on top of the topographically arranged retinal disparity input units. Note that the population tuning for retinal disparity is non-symmetric, in contrast to the symmetric (Gaussian) tuning for retinal positions.

#### Eye-in-head, head-on-body and vergence inputs

The 3D visuomotor transformation depends critically on extraretinal information about body geometry as encoded by eye-in-head and head-on-body signals [1] and on fixation distance as coded by ocular vergence [46]. Eye and head orientations were coded as angular vectors in a 6D push-pull antagonistic arrangement inspired by motor neuron activity [16], [82], [83], [84]. Angular vectors (r_X_, r_Y_, r_Z_) equal to the unitary rotation axis multiplied by the rotation angle in degrees. This results in a unique angular vector describing the shortest path of rotation from one point to another in 3-dimensional space (the negative of a given angular vector describes the exact opposite rotation of the positive counter-part). The 3D angular vectors for eye and head orientation were then transformed into two 6D arrays of inputs (one for eye and one for head orientation) as follows [13], [76], [85]:
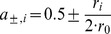
(5)where the maximum angle of rotation, r_0_, was equal to 50deg for eye and 70deg for head orientations (the data set included rotations up to 45deg for eye and 65deg for head orientations). Also, the coordinate system for encoding eye orientation angular vectors was rotated 45deg around the z-axis (i.e. vertical axis) to reproduce the mixed vertical-torsion encoding of eye orientations in the brainstem neural integrator [86], [87], [88], [89]. As a result, eye and head orientation units show linear increases/decreases of activity for orientations away from straight-ahead. For example for a 25deg horizontal eye orientation, the two units coding for horizontal orientation would be at 0.25 and 0.75, while the other four units (coding for vertical and torsional orientations) would be at 0.5.

To encode the ocular vergence angle, we used a single positive input. The vergence angle ϕ_V_ was defined as the absolute angle between the right and left eye gaze directions (in degrees), so that larger vergence angles represent closer fixations. The activation of the vergence unit changed linearly with vergence angle (0deg vergence corresponded zero activation) and was determined as follows:
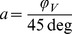
(6)


Note that previous studies have used both distributed [10], [11], [14], [15], [16] and lumped [13], [42], [76], [85] codes for eye/head/vergence signals. However, they all found qualitatively the same (gain-modulated) behavior in their hidden layer units. Therefore, we chose to use a lumped code for simplicity.

### Population output units and network output

The network output (4^th^ layer) consisted in 3 units that coded the movement vector in Euclidean space, each unit coding movement distance along one spatial direction, i.e. cardinal axes X (horizontal), Y (posterior-anterior) and Z (vertical). These output units were designed to decode the distributed representation of the movement vector encoded by the population output units (POUs, 3^rd^ layer) and act as a behavioral read-out allowing for an unambiguous quantitative interpretation of single POU unit activity [10]. For that purpose, we calculated the connection weights between layers 3 and 4 prior to training based on the assumption of cosine tuning in layer 3 (see below) and kept those weights unaltered during network training.

We used 125 cosine-tuned POUs in our network with preferred movement directions (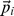) randomly and uniformly distributed on a unit sphere [13], [90], [91], as shown in Figure 1. We used a statistically uniform distribution of 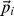 to match the above-cited electrophysiological findings. Cosine-tuned neurons that encode movement direction in intrinsic, effector-centered coordinates have been observed in pre-motor cortex of the monkey [8], [38], [39], [40] and theoretically, cosine tuning is optimal for motor control in 3D [4], [64]. To compute the behavioral read-out weights, we assumed cosine tuning for each POU i, such that
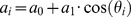
(7)where a_0_ = 0.5 is the baseline firing rate and 
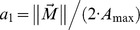
 is the cosine scaling parameter that scaled the unit activity to the size of the required movement 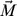, whereas the angle 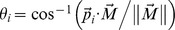 coded for movement direction [92], [93]. The maximum movement amplitude was A_max_ = 2m (maximum possible movement in the data set: 175cm), resulting in a_i_ ∈ [0,1]. Note that we did not train the network to reproduce the theoretical activations specified in Eq. 7; however, indirectly the read-out of POUs (see below) might have enforced cosine tuning, as previously shown [13].

The assumption of cosine tuning in for POUs (layer 3) allowed us to explicitly compute the optimal read-out weights w_ij_ between layers 3 and 4 using an optimal linear estimator, OLE [94], such that
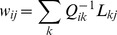
(8)


In Eq. 8, j stands for the vector component, i.e. X, Y or Z. For full cosine tuning (Eq. 7), the center of mass matrix L_kj_ and the cross-correlation matrix Q_ik_ can be calculated as [13]:
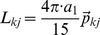
(9)

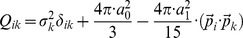
(10)


The cross-correlation matrix Q_ik_ contains an estimate of the expected neural noise (σ_k_ arbitrarily set to 0.01) and a dot product that specifies the interaction between two tuning curves. We chose the expected neural noise and number of POU units based on the theoretical read-out precision, which was an average read-out error <2cm [13]. Again, once the read-out weights were computed, they were held constant during the training process of the network. Also, we did not constrain the activation of the POUs in any way. Finally, the choice of a uniform distribution of POU preferred directions did not affect or constrain the read-out process, since OLE works with arbitrary distributions.

### Training method and training set

To train our network, we used a training set computed from an exact geometrical model of the 3D eye-head-shoulder-hand linkage [1]. This model computed 3D binocular eye positions that obeyed binocular Listing's law [95], [96], [97], [98], which constrains the 3 degrees of freedom (dof) of each eye's rotation to 2 effective dof. This binocular version of Listing's law is modulated by the static vestibulo-ocular reflex, i.e. VOR induces ocular counter-roll for head roll and a tilt of Listing's plane with head pitch [99], [100]. It was important to include binocular Listing's law including the VOR modulations because this results in different retinal disparity pattern depending on eye-head orientations [46] that need to be interpreted correctly to compute reach depth.

For the training set, eye and head orientations were approximately uniformly distributed and fixation distance varied between 25cm and 5m so that vergence was approximately uniformly distributed. Hand and target positions were randomly chosen with reach space (not more than 85cm from the right shoulder) and in a way that neither exceeded 70deg visual eccentricity. We then computed the projections of hand and target positions onto each eye (used to compute retinal disparity) and onto a hypothetical cyclopean eye (this was the retinal position input) and also calculated the 3D reach plan in effector-centered coordinates.

We trained a 200 hidden layer unit (HLU – 2^nd^ layer) network using 125 population output units (POU – 3^rd^ layer). We also trained various other networks with different number of HLUs (9, 16, 25, 36, 49, 64, 81 and 100 HLUs) yielding qualitatively the same results and we therefore only concentrated on the largest network, i.e. 200 HLU network. The neural network was implemented in Matlab R2007a (Mathworks Inc, Natick, MA) using the neural network toolbox and customized functions. Batch training was performed through a pseudo-Newton method with preconditioned conjugate gradient descent [101], [102], [103] using 250,000 training points and was stopped arbitrarily at 10,000 iterations.

### Network analysis

To quantify whether the network used the extraretinal signals for the 3D visuomotor transformation, we computed a 3D compensation index as the regression slope between the predicted and observed 3D compensation. The predicted (observed) 3D compensation was the difference between the ideal (actual) movement vector and the movement vector that would have resulted if all extra-retinal signals were ignored [1], [13]. We also computed sensitivity vectors, which are determined by the network weights and indicate in which direction a particular network input modulates a given unit most strongly. For example, the vergence sensitivity vector of a HLU was determined by the projection strength (weight) between the vergence input and the HLU. For a given POU, the vergence input weights were multiplied by the in-between layer weights connecting to the POU (dot product), resulting in a scalar vergence sensitivity vector.

Receptive field (or disparity tuning) shift indices in Figures 4, 5, 8, 9 and 11 were computed as the regression slope between the centre-of-mass and the variable under investigation, such as previously done [13]. For example, for vergence-related RF shifts (Figure 4), we computed the centre-of-mass (in angular position, i.e. degrees) of the visual receptive field of a unit for different vergence angles. Systematically changing the vergence angle in 1 deg steps resulted in a series of RF positions that we then regressed against the corresponding vergence angles. The slope of this regression is then the vergence-related RF shift gain. We proceeded in equivalent ways for the other analyses.

Gain modulation indices in Figure 6 were computed similarly to Bhattacharyya et al. [26]. We determined each network unit's activation for every combination of vergence, hand and target depth using 4 different vergence angles and 5 different hand/target depths, all within reach. We then computed the gain modulation index as: 
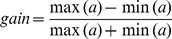
, where *a* was for example the activity of a unit across all vergence angles (in case of the vergence gain modulation). We calculated this gain for all combinations of unrelated parameters (e.g. hand/target depth in case of vergence gain modulation) and then averaged the result to obtain one single gain value for each unit and each modulation dimension. We chose an arbitrary threshold of 0.2 to determine whether a unit was gain modulated or not; this threshold is qualitatively similar to ones used in electrophysiological studies [26].
